# Genome Sequence of *Lactobacillus rhamnosus* Strain CNCM I-3698

**DOI:** 10.1128/genomeA.00582-15

**Published:** 2015-06-11

**Authors:** R. Tareb, M. Bernardeau, J. P. Vernoux

**Affiliations:** a Research Unit EA 4651 Aliments Bioprocédés Toxicologie Environnements (ABTE), IFR146 ICORE, Université de Caen, Basse-Normandie, Caen, France; b DuPont, Industrial Biosciences, Danisco Animal Nutrition, Marlborough, United Kingdom

## Abstract

*Lactobacillus rhamnosus* CNCM I-3698 is a commercially available probiotic that is used in animal feed as an additive. Here, we announce the draft genome sequence for this strain, consisting of 71 contigs corresponding to 2,966,480 bp and a G+C content of 46.69%.

## GENOME ANNOUNCEMENT

Lactobacilli colonize the human and animal gastrointestinal tract as normal flora (1), and they can regulate the intestinal homeostasis by providing several beneficial properties, including pathogen inhibition and immunomodulation (1–4). *Lactobacillus rhamnosus* CNCM I-3698 was isolated from goat rumen. It is a probiotic strain having a scientific and commercial interest. It is currently used as a feed additive in Europe (5). Antagonistic activities against zoonotic pathogens of swine, such as *Brachyspira* spp. (6) and *Campylobacter* spp. (7), have been demonstrated *in vitro*, most likely through aggregation and adhesion exclusion properties. Antimicrobial and zootechnical properties have been demonstrated *in vivo* in pigs (8).

The uncompleted draft genome sequence of *L. rhamnosus* CNCM I-3698 was determined by paired-end sequencing using the Illumina GAIIx platform (BaseClear, the Netherlands), with a 100-bp paired-end library and a coverage of >100×. The reads were assembled *de novo* into 71 contigs using CLC Genomics Workbench 5.0 (CLC bio, Denmark). The functional annotation of predicted genes was achieved using the RAST server (9) and the NCBI Prokaryotic Genome Annotation Pipeline (PGAP).

The draft genome of *L. rhamnosus* CNCM I-3698 includes 2,966,480 bp, with a G+C content of 46.69%. *L. rhamnosus* CNCM I-3698 contains 2,939 predicted protein-coding genes and 55 RNA coding regions. Among all predicted protein-coding genes, we assigned 2,014 (68.5%) to known protein functions, with 925 (31.5%) remaining as hypothetical proteins. There are 328 subsystems that are represented in the genome, and we used this information to reconstruct the metabolic network (determined using the RAST server).

Preliminary comparative analysis using the MUMmer method (10) and genomic BLAST (http://www.ncbi.nlm.nih.gov/sutils/genom_table.cgi) (11) of *L. rhamnosus* CNCM I-3698 and four other currently available *L. rhamnosus* complete genome sequences (http://www.ncbi.nlm.nih.gov/genome/) showed that *L. rhamnosus* CNCM I-3698 is of low similarity to the probiotic isolates *L. rhamnosus* GG substrain Helsinki and substrain Tokyo (query cover, 87%; 100% maximum identity). In contrast, *L. rhamnosus* CNCM I-3698 is highly similar to the dairy industry isolates *L. rhamnosus* Lc 705 (query cover, 97%; 100% maximum identity) and *L. rhamnosus* ATCC 8530 (query cover, 97%; 100% maximum identity).

Genome analysis identified the presence of genes encoding proteins for a variety of cell surface proteins involved in cell aggregation, biofilm formation, and adherence, which may play an important role in the colonization of the gastrointestinal tract. The genome contains coding sequences related to fibronectin/fibrinogen binding protein; translation elongation factor Tu; mucus binding factor and adhesin involved in aggregation, adhesion, and biofilm formation; lipoprotein; lipoteichoic acids; a bacteriocin biosynthetic protein; and a bile salt hydrolase. Those functions likely contribute to the gastric survival of the strain and promote its interactions with the intestinal mucosa and microbiota, such as competitive exclusion of pathogenic bacteria and modulation of the immune system (12, 13).

A more detailed analysis aiming at evaluating the stability, safety, functional, and metabolic aspects of this strain is in progress.

### Nucleotide sequence accession numbers. 

This whole-genome shotgun project has been deposited at DDBJ/EMBL/GenBank under the accession no. LAZE00000000. The version described in this paper is version LAZE01000000.
